# Targeting CRMP2 for Chronic Pain: From Molecular Mechanisms to Therapeutic Strategies

**DOI:** 10.3390/biomedicines14071512

**Published:** 2026-07-05

**Authors:** Jia-Yi Wang, Dai-Qiang Liu, Ya-Qun Zhou, Wei Mei

**Affiliations:** Department of Anesthesiology and Pain Medicine, Hubei Key Laboratory of Geriatric Anesthesia and Perioperative Brain Health, and Wuhan Clinical Research Center for Geriatric Anesthesia, Tongji Hospital, Tongji Medical College, Huazhong University of Science and Technology, Wuhan 430030, China

**Keywords:** CRMP2, chronic pain, synaptic plasticity, neuroimmune interaction

## Abstract

Collapsin Response Mediator Protein 2 (CRMP2) has emerged as a central node in the pathogenesis of chronic pain, functioning as a multimodal ‘molecular switch’ that regulates microtubule dynamics, ion channel trafficking, and synaptic plasticity. The dysregulation of CRMP2, particularly through aberrant post-translational modifications (PTMs) such as phosphorylation and SUMOylation, is a critical driver of both peripheral and central sensitization. This review systematically examines the structure, regulation, and multifaceted roles of CRMP2 in pain signaling pathways. We then critically evaluate a spectrum of CRMP2-targeted therapeutic strategies, including small-molecule inhibitors, peptide-based agents, and gene silencing, highlighting their promising preclinical efficacy and safety profiles. Despite challenges in targeting specificity and central nervous system delivery, we posit that innovations in delivery systems, precision medicine, and AI-assisted drug design will catalyze the clinical translation of CRMP2-based, non-opioid analgesics, offering a paradigm shift in chronic pain management.

## 1. Introduction

Chronic pain, a debilitating global health issue affecting hundreds of millions, is now recognized not merely as a symptom but as a distinct disease entity in modern medicine [1]. Chronic pain affects approximately 20–30% of adults worldwide, and its prevalence continues to increase with population aging and the growing burden of chronic diseases [2]. This condition severely compromises patients’ quality of life, leading to functional disability, anxiety, depression, and opioid dependence, while imposing substantial socioeconomic burdens [3]. Current first-line treatments, including non-steroidal anti-inflammatory drugs (NSAIDs) and opioids, present significant clinical challenges due to limited efficacy, addiction potential, tolerance, and severe systemic side effects [4]. Consequently, a deeper understanding of the molecular mechanisms driving the onset and persistence of chronic pain is imperative for developing innovative, effective, and well-tolerated precision analgesic strategies, addressing a critical ‘unmet clinical need’ [5].

Originally identified for its role in neuronal growth cone collapse, CRMP2 is the most extensively characterized member of the CRMP family. This cytoplasmic phosphoprotein plays a pivotal role in the nervous system, governing processes such as neuronal differentiation, axon guidance, dendrite formation, and synaptic plasticity [6]. Its biological functions are tightly regulated by PTMs, with phosphorylation status being of particular importance. CRMP2 regulates microtubule assembly and dynamics through interactions with tubulin and multiple signaling partners. Under physiological conditions, CRMP2 is a key regulator of central nervous system (CNS) development and homeostasis.

Accumulating evidence now links CRMP2 dysfunction to the pathology of various neurological disorders, including chronic pain. Significant alterations in CRMP2 expression and phosphorylation status have been observed in multiple chronic pain models, such as neuropathic and inflammatory pain [7,8]. Beyond its structural roles, CRMP2 acts as a multifunctional signaling hub within pain pathways [9]. It directly regulates the membrane trafficking and activity of N-type voltage-gated calcium channels (Cav2.2), thereby influencing neurotransmitter release from nociceptive sensory neurons [10]. Additionally, CRMP2 modulates neuronal excitability by affecting sodium channels (e.g., Nav1.7) [11], and participates in the signal transduction of neurotrophic factors like BDNF, as well as synaptic transmission regulation. These findings collectively position CRMP2 as a master regulator of pain sensitization, rendering it an attractive novel target for analgesic development. Neuron-specific inhibition of CRMP2 has shown analgesic effects in preclinical neuropathic pain models while largely preserving physiological nociceptive responses [12].

Although previous reviews have addressed selected aspects of CRMP2 in pain [13,14], a focused synthesis that integrates phosphorylation, SUMOylation, NaV1.7/CaV2.2 trafficking, synaptic plasticity, neuroimmune interfaces, and translational CRMP2-targeted analgesic strategies in chronic pain remains limited. This review is therefore important because it consolidates mechanistic and therapeutic evidence into a pain-centered framework. This review provides three practical outputs: (i) a mechanistic map linking CRMP2 post-translational modifications to peripheral and central sensitization; (ii) a model-by-model summary of preclinical evidence and intervention strategies; and (iii) a translational appraisal of target specificity, delivery barriers, safety liabilities, and future study priorities for non-opioid analgesic development.

## 2. Search Strategy and Literature Selection Criteria

This article is a narrative review. Literature was identified through searches of PubMed/MEDLINE, Web of Science, Scopus and Embase up to June 2026. The search strategy combined CRMP2-related terms (“CRMP2”, “collapsin response mediator protein 2”) with pain-related terms (“chronic pain”, “neuropathic pain”, “inflammatory pain”, “postoperative pain”, and “osteoarthritis pain”).

Studies were included if they met at least one of the following criteria: (i) original experimental evidence linking CRMP2 expression, localization, phosphorylation, SUMOylation, or protein–protein interactions to pain-related signaling or behavior; (ii) preclinical intervention studies targeting CRMP2, CRMP2-Ubc9, CRMP2-CaV2.2, CRMP2-Nav1.7, or CRMP2 phosphorylation/deSUMOylation pathways; (iii) clinical or translational studies relevant to CRMP2 autoantibodies, drug repurposing, gene therapy, or delivery barriers; or (iv) high-quality reviews providing mechanistic or structural context. Exclusion criteria were duplicate reports, non-English articles, studies focused only on non-CRMP2 CRMP family members unless CRMP2-specific data were provided, papers unrelated to pain, and studies without interpretable mechanistic or behavioral outcomes.

## 3. An Overview of CRMP2: Structure, Function, and Regulation

### 3.1. Molecular Structure

**Figure 1:**
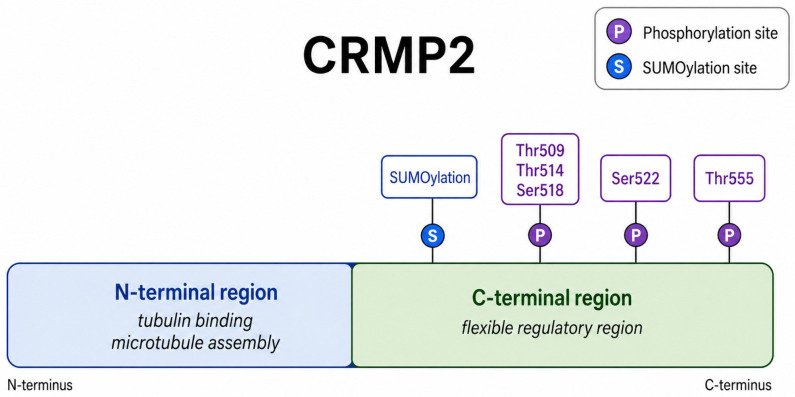
Representative schematic structure of CRMP2.

CRMP2 is a key member of the CRMP family (CRMP1–5). Structurally, CRMP2 contains multiple functional regions that support its interactions with diverse protein partners. The N-terminal region contributes to tubulin binding and microtubule assembly, whereas the C-terminal region is relatively flexible and contains several regulatory residues that are subject to post-translational modifications, including phosphorylation and SUMOylation. CRMP2 can form homo- or hetero-oligomeric complexes, which further expands its capacity to regulate cytoskeletal dynamics, ion-channel trafficking, and synaptic function. This multi-domain and highly regulated structural organization underlie the diverse biological functions of CRMP2 in neuronal development and pain-related signaling pathways [15]. A representative schematic structure of CRMP2 is shown in Figure 1.

### 3.2. Canonical Function: Cytoskeletal Regulation and Neuronal Polarity

The most established function of CRMP2 is the dynamic regulation of the cytoskeleton. It binds to tubulin heterodimers, thereby promoting microtubule assembly, which is essential for axon elongation and neurite outgrowth [15,16]. During neuronal development, CRMP2 is central to establishing and maintaining neuronal polarity (i.e., the distinct identities of axons and dendrites) by orchestrating tubulin heterodimer transport, microtubule assembly, growth-cone dynamics, and polarity-related endocytic trafficking.

### 3.3. Non-Canonical Functions: Direct Regulation of Ion Channels

Beyond cytoskeletal regulation, a core non-canonical function of CRMP2 is the direct modulation of ion channel activity. Research demonstrates that CRMP2 directly interacts with voltage-gated calcium channels (e.g., Cav2.2) and voltage-gated sodium channels (e.g., Nav1.7), influencing their membrane localization, and stability, thereby rapidly modulating neuronal excitability [10,11,17].

### 3.4. Regulatory Mechanisms: Post-Translational Modifications (PTMs)

The functional activity, subcellular localization, and protein–protein interactions of CRMP2 are precisely controlled by PTMs, with phosphorylation and SUMOylation being the most extensively studied.

Phosphorylation: Multiple kinases, including Cyclin-dependent kinase 5 (CDK5), Glycogen synthase kinase 3 beta (GSK3β), and Rho-associated protein kinase (ROCK), phosphorylate CRMP2 at specific residues (e.g., Ser522, Thr509/514/518, Thr555). These phosphorylation events alter its affinity for tubulin and regulate CRMP2-dependent ion channel function [18,19,20].

SUMOylation: CRMP2 can be modified by Small Ubiquitin-like Modifier (SUMO) proteins. This modification, catalyzed by the E2 conjugating enzyme Ubc9, critically regulates the interaction between CRMP2 and the Nav1.7 channel, thereby influencing channel trafficking and current amplitude [7,11].

## 4. Aberrant Regulation of CRMP2 in Chronic Pain

Under chronic pain conditions, the expression, subcellular distribution, and PTM status of CRMP2 undergo profound changes. These aberrant regulatory events are key molecular processes that contribute to the persistent amplification of pain signals. Emerging evidence has demonstrated dysregulated CRMP2 expression and activation in the dorsal root ganglia (DRG) and spinal cord dorsal horn in rodent models of chronic pain (Table 1). However, current evidence does not support a definitive conclusion that specific rodent strains independently modify CRMP2 expression. Therefore, CRMP2 alterations should be interpreted mainly according to the pain model, tissue region, and time point examined.

### 4.1. Altered Expression Levels and Localization

In neuropathic pain models such as spinal nerve ligation (SNL), and spared nerve injury (SNI), CRMP2 expression has been reported to change in a model-dependent manner. More importantly, CRMP2’s subcellular localization shifts becoming enriched in presynaptic terminals, consistent with its role in enhancing neurotransmitter release.

### 4.2. Dysregulation of PTMs

The most prominent feature of CRMP2 dysregulation in pain states is the alteration of its phosphorylation and SUMOylation levels.

Enhanced Phosphorylation: Following nerve injury, CDK5-mediated phosphorylation of CRMP2 at Ser522 is significantly increased. This ‘priming’ phosphorylation is a critical step for the functional upregulation of CRMP2 in neuropathic pain [18,21]. Subsequent phosphorylation by GSK3β further modifies CRMP2 activity and may contribute to abnormal ion channel regulation in chronic pain [19,28].

Elevated SUMOylation Levels: In the rat SNI model, CRMP2 SUMOylation levels are significantly elevated in the ipsilateral spinal cord dorsal horn, while total protein levels remain unchanged. This increase correlates with enhanced synaptic localization of Nav1.7, suggesting that CRMP2 SUMOylation promotes Nav1.7 membrane trafficking [7].

Collectively, these aberrant PTMs shift CRMP2 from its role in maintaining cytoskeletal stability to a ‘pro-nociceptive’ mode that preferentially binds ion channels, enhancing neuronal excitability and synaptic transmission.

## 5. The Role of CRMP2 in the Mechanism of Chronic Pain

**Figure 2:**
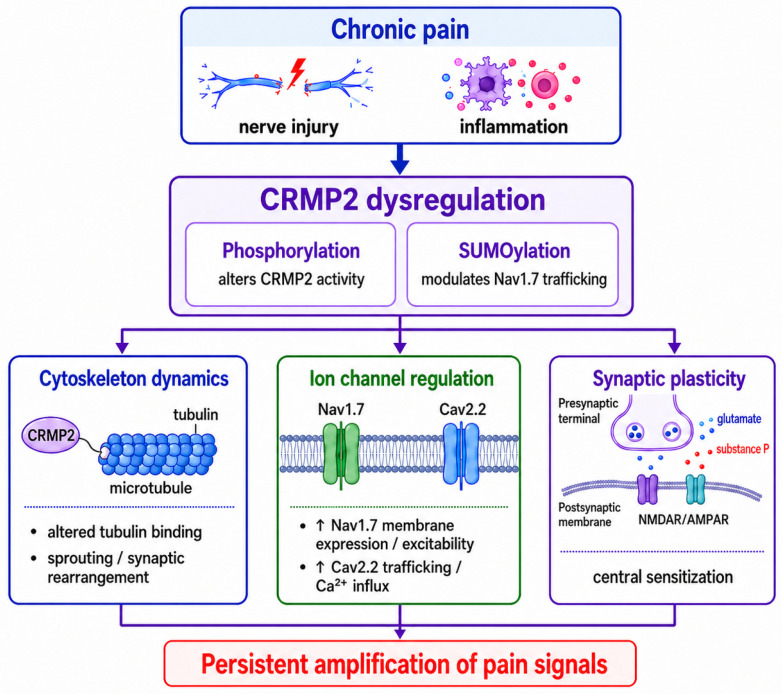
Core mechanism of CRMP2 dysregulation in chronic pain. ↑: upregulation.

CRMP2 has evolved from a developmental protein to a central node in the molecular network underlying pain signaling. Its dual role in regulating both the cytoskeleton and key ion channels allows it to control both the structural and functional plasticity of neurons in pain pathways (Figure 2).

### 5.1. CRMP2 and Cytoskeleton Dynamics

In pathological pain, cytoskeletal remodeling is not limited to the developmental stage. After nerve injury or inflammation, adult sensory neurons undergo phenotypic changes and initiate processes of regenerative sprouting and synaptic rearrangement—these processes depend on dynamic cytoskeletal changes. CRMP2 is believed to drive this structural plasticity through its tubulin-binding activity, ultimately leading to neuronal hyperexcitability and abnormal pain signal transmission [26]. For example, phosphorylated CRMP2 (e.g., pCRMP2-Ser522) has reduced affinity for tubulin, thereby “detaching” it from cytoskeleton-related functions and redirecting it to participate in ion channel regulation. Additionally, in the spared nerve injury (SNI) model, the expression of CRMP2 phosphorylated by Cdk5 is upregulated in specific neuronal subpopulations, further promoting cytoskeletal reorganization and pain behaviors [22].

### 5.2. CRMP2 and Ion Channel Regulation

Voltage-Gated Calcium Channels (VGCCs): CRMP2 can directly bind to the cytoplasmic domain of N-type voltage-gated calcium channels (Cav2.2), and this interaction is crucial for the trafficking of Cav2.2 channels to the neuronal plasma membrane [10,17,29]. After the channels reach the membrane, binding with CRMP2 enhances channel activity, increasing calcium influx during depolarization. Since presynaptic Cav2.2 channels play a key role in regulating the release of pain neurotransmitters (such as glutamate and substance P) in the spinal cord, CRMP2-mediated enhancement of Cav2.2 function significantly promotes the transmission of pain signals from the periphery to the CNS [10,17,29].

Voltage-Gated Sodium Channels (VGSCs): CRMP2 also interacts with the Nav1.7 sodium channel, a key channel closely associated with human pain disorders [11,30]. This interaction increases the membrane expression density and current amplitude of Nav1.7. As Nav1.7 determines the action potential generation threshold of nociceptors, CRMP2-mediated upregulation of its expression significantly lowers the neuronal activation threshold, increases the firing frequency of nociceptive neurons, and ultimately leads to hyperalgesia and allodynia [31,32]. Further studies have revealed that there is a unique CRMP2-binding domain (CRS) in the first intracellular loop of Nav1.7, which is absent in other sodium channel subtypes [33]. This specificity enables targeted interference with the CRMP2–Nav1.7 interaction without affecting the function of other sodium channels. Notably, in chronic pain models, the binding of CRMP2 to the CRS domain of Nav1.7 is enhanced [31,33].

Taken together, the CRMP2–Nav1.7 and CRMP2–Cav2.2 axes represent the most translationally relevant mechanisms linking CRMP2 dysregulation to chronic pain. Nav1.7 primarily controls the excitability threshold of peripheral nociceptors, whereas Cav2.2 governs presynaptic calcium influx and neurotransmitter release in the spinal dorsal horn. Therefore, CRMP2 is not merely a cytoskeletal regulatory protein but an upstream coordinator that couples peripheral neuronal excitability with central synaptic transmission.

### 5.3. CRMP2 and Post-Translational Modifications (PTMs)

#### 5.3.1. Phosphorylation

CDK5-Mediated Phosphorylation: The phosphorylation state of CRMP2 plays a critical role in neuropathic pain, especially in specific subpopulations of dorsal root ganglion (DRG) neurons. CDK5 phosphorylates CRMP2 at the Ser522 site, and this “priming” phosphorylation is a key step in the upregulation of CRMP2 function in neuropathic pain models [21,22]. CRMP2 phosphorylated at Ser522 (pCRMP2-Ser522) has reduced affinity for tubulin, and its function shifts from cytoskeleton regulation to enhancing binding with Cav2.2 channels, thereby promoting channel trafficking and nociceptive signal transmission [22]. After nerve injury (SNI model), the level of CDK5-mediated CRMP2 phosphorylation at Ser522 in the spinal dorsal horn and DRG is significantly increased, with enrichment in presynaptic regions [22,24].

#### 5.3.2. Ubiquitin-like Modification (SUMOylation)

In addition to phosphorylation, SUMOylation of CRMP2 is also involved in pain regulation. In the rat spared nerve injury (SNI) model, the SUMOylation level of CRMP2 in the ipsilateral dorsal horn of the spinal cord is significantly increased, which is associated with enhanced synaptic localization of Nav1.7. This suggests that SUMOylation of CRMP2 can promote the membrane trafficking of Nav1.7, thereby enhancing nociceptive signal transmission [7,34]. Interfering with CRMP2 SUMOylation can selectively reduce Nav1.7 current and reverse experimental neuropathic pain [7,31,35]. Studies on transgenic mice with CRMP2 SUMOylation deficiency have further revealed its sex-specific regulatory role in chronic neuropathic pain [34].

### 5.4. CRMP2 and Synaptic Plasticity

A core mechanism of chronic pain is central sensitization, which is a form of synaptic plasticity. By regulating presynaptic Cav2.2 channels to affect neurotransmitter release and modulating receptor function through postsynaptic mechanisms (e.g., regulating the expression of the NR2B subunit of NMDA receptors), CRMP2 participates in the regulation of synaptic plasticity [23,24]. In this context, CRMP2 may serve as a molecular bridge linking peripheral nociceptor hyperexcitability with central synaptic amplification. At the presynaptic level, CRMP2-mediated facilitation of Cav2.2 trafficking and activity can enhance calcium-dependent release of excitatory neurotransmitters, including glutamate and substance P, from primary afferent terminals. Studies have shown that inhibiting the phosphorylation of CRMP2 can reduce the expression of AMPA receptors and the NR2B subunit of NMDA receptors, and regulate synaptic plasticity [23]. Insular cortex stimulation (ICS) exerts an analgesic effect by inhibiting the phosphorylation of CRMP2 at the Thr514 site, reducing the expression of AMPA receptors and NR2B [23]. Therefore, CRMP2-related interventions may not only attenuate peripheral sensitization but also reduce central sensitization by disrupting maladaptive neurotransmitter release and receptor-dependent synaptic plasticity.

### 5.5. CRMP2 and Neuroimmune Interaction

Although CRMP2 is mainly expressed in neurons, emerging evidence suggests that it may be involved in neuroimmune interactions. In neuropathic pain models, post-translational modifications of CRMP2 at the spinal cord level undergo significant changes, and the spinal cord is a hub for neuron-glial cell crosstalk [36]. More importantly, CRMP2 autoantibodies have been detected in patients with spinal cord injury, and these antibodies are associated with the development of neuropathic pain—this strongly suggests that CRMP2 may act as an immunogen to participate in the chronic neuroinflammatory process [36]. However, direct evidence linking CRMP2 to microglial activation, astrocyte signaling, cytokine regulation, mitochondrial dysfunction, oxidative stress, or specific neuroinflammatory cascades remains limited. At present, it is still unclear whether CRMP2 directly regulates glial function or whether CRMP2-related changes occur secondarily to neuronal injury and persistent nociceptive input. Therefore, future studies should determine whether CRMP2 dysregulation participates causally in neuron-glia communication and neuroinflammation, or whether it mainly reflects broader injury-associated stress responses. Although research in this field is still in its early stages, it provides a new perspective for understanding the comprehensive role of CRMP2 in pain.

## 6. Therapeutic Strategies Targeting CRMP2

In this review, we have summarized the abnormal expression of CRMP2 in chronic pain. Furthermore, we have discussed the therapeutic potential of targeting CRMP2 for chronic pain in preclinical studies (Table 2).

### 6.1. Small-Molecule Inhibitors

Due to their favorable druggability and oral bioavailability, small-molecule inhibitors are the preferred strategy for drug development. These drugs mainly exert their effects by disrupting the protein–protein interactions (PPIs) between CRMP2 and ion channels.

Lacosamide Enantiomers and Optimized Derivatives: The most representative example is the (S)-enantiomer of the antiepileptic drug lacosamide. Early studies suggested that its analgesic effect originated from the slow inactivation of sodium channels, but subsequent research revealed a unique mechanism—direct binding to CRMP2 [9,29,40]. (S)-lacosamide can specifically disrupt the interaction between CRMP2 and the cytoplasmic tail region of Cav2.2 channels, reducing the accumulation of channels in the presynaptic membrane [29,40]. This effect inhibits the release of nociceptive neurotransmitters (such as glutamate and substance P), ultimately weakening pain signal transmission [41]. However, its relatively moderate efficacy and the inactivity of its stereoisomer (R)-lacosamide limit its clinical application.

Compound 194: Compound 194 is a small-molecule inhibitor that can block the interaction between CRMP2 and Ubc9, thereby inhibiting CRMP2 SUMOylation and reducing the membrane localization and current of Nav1.7 [31,32]. It has high selectivity for Nav1.7 and does not affect other sodium channel subtypes (Nav1.1–1.6, 1.8–1.9) or the cardiac hERG potassium channel [31]. Oral administration of Compound 194 can significantly reverse mechanical allodynia in rats with chronic constriction injury (CCI) and reduce pain-related behaviors in the mechanical conflict avoidance (MCA) test [37]. Notably, its effect is selective—it is only effective in pain models, has no impact on normal animals, and exhibits good safety [31,37]. In addition, Compound 194 can reduce the excitability of trigeminal ganglion (TG) neurons and effectively alleviate trigeminal neuralgia; intranasal administration, as a non-invasive and effective drug delivery route, provides a new strategy for the treatment of craniofacial pain [39]. By indirectly inhibiting Nav1.7 channels, Compound 194 can also reduce pain behaviors and related neuronal abnormalities in osteoarthritis (OA) models, providing a new potential target for OA pain treatment [32]. This indirect targeting strategy circumvents the challenges of direct Nav1.7 inhibition and demonstrates clinical translation potential [31,32].

NMDA Receptor-Targeting Inhibitors: In addition, small-molecule inhibitors such as PEAQX (a highly selective competitive antagonist for NMDA receptors containing the NR2A subunit) and ifenprodil (a selective non-competitive allosteric inhibitor for NMDA receptors containing the NR2B subunit) can mimic the effect of insular cortex stimulation (ICS) by selectively inhibiting the NR2A or NR2B subunit of NMDA receptors [23]. ICS exerts an analgesic effect by inhibiting the phosphorylation of CRMP2 at the Thr514 site, reducing the expression of AMPA receptors and the NR2B subunit of NMDA receptors, and regulating synaptic plasticity [23].

### 6.2. Peptide Inhibitors and Peptidomimetics

By mimicking specific short peptide sequences of CRMP2 or its binding partners, peptide inhibitors competitively block protein–protein interactions with high affinity and specificity.

Targeting the CRMP2–Cav2.2 Binding Domain: A well-established strategy is to disrupt the interaction between CRMP2 and the presynaptic Cav2.2 channel complex. Early studies designed competitive inhibitory peptides based on the CRMP2-derived Cav2.2-binding domain 3 (CBD3), a short sequence that mediates the CRMP2–Cav2.2 interaction. Brittain et al. developed the TAT-fused CBD3 peptide (TAT-CBD3), which contains a cell-penetrating TAT motif, and demonstrated that uncoupling CRMP2 from the presynaptic Cav2.2 channel complex suppresses both inflammatory and neuropathic pain behaviors [44]. This study provided the first in vivo evidence that targeting the CRMP2–Cav2.2 interaction has analgesic potential.

To improve the stability, efficacy, and specificity of lead peptides, subsequent studies have conducted optimization in multiple aspects. Through alanine scanning mutagenesis of TAT-CBD3, an optimized variant TAT-CBD3-A6K was screened [42]. This variant not only provides sustained analgesic effects in neuropathic pain models but, more importantly, has no opioid-like addiction potential in drug discrimination experiments, improving safety [42]. In addition, researchers have developed N-myristoylated CRMP2 peptides (Myr-TAT-CBD3) [41]. This modification anchors the peptide to the cell membrane, forming a “membrane-restricted” local high-concentration environment, thereby efficiently inhibiting the membrane trafficking and function of Cav2.2 and successfully reversing inflammatory pain and postoperative pain behaviors [41].

#### 6.2.1. From Peptides to Peptidomimetics

Although peptide inhibitors exhibit certain efficacy, their inherent limitations hinder clinical translation. To address this issue, research has shifted toward the development of small-molecule peptidomimetics with better druggability. The Khanna team converted the active sequence of the CBD3 peptide into a small-molecule peptidomimetic CBD3063 through structure-based drug design. This molecule can effectively disrupt the CRMP2–Cav2.2 interaction and exhibit potent and sustained analgesic effects in various chronic pain models, representing a key step toward clinical application [45].

Mechanism Deepening and Target Expansion: Research progress has further revealed the regulatory network of CRMP2. In addition to directly targeting the CRMP2–Cav2.2 axis, other related protein interactions have also been identified [13,14]. One review systematically challenges the dogma of traditional chronic pain treatment and discusses the broad prospects of peptide strategies targeting CRMP2 and its multiple interaction partners (e.g., Cav2.2, tubulin) [14]. Another review specifically summarizes the design principles and analgesic effects of various peptide and peptidomimetic inhibitors targeting the CRMP2–Cav2.2 interaction [13].

In addition, the application scope of targeting strategies is expanding. In preclinical joint pain models, disrupting the CRMP2–Cav2.2 interaction using TAT-CBD3 can effectively reduce pain-like behaviors, providing new insights for the treatment of chronic joint pain such as osteoarthritis [8]. Furthermore, CRMP2 forms a functional complex with neurofibromin, which also regulates Cav2.2 and pain behaviors [46]. The designed cell-penetrating peptide (TAT-NF1) can specifically disrupt this complex, producing an analgesic effect and revealing a new mechanism of CRMP2-mediated pain signal transmission and potential drug targets [27,46,47,48].

#### 6.2.2. Targeting the CRMP2–Nav1.7 Interaction

Although Nav1.7 is a key target for pain treatment, direct inhibition of this channel has shown poor efficacy in clinical trials. Replacing the CRS domain of Nav1.7 with the homologous sequence of other sodium channels can significantly reduce Nav1.7 current [33]. Using a cell-penetrating peptide (Myr-TAT-Nav1.7-CRS) to competitively inhibit CRMP2–Nav1.7 binding can reduce the membrane localization and current of Nav1.7 without affecting other sodium channels or physiological pain [33].

Targeting CRMP2 SUMOylation: Interfering with CRMP2 SUMOylation can also selectively reduce Nav1.7 current and reverse experimental neuropathic pain [7,35]. The t-CSM peptide is a cell-penetrating peptide designed based on the SUMOylation motif (CSM) of CRMP2, which can effectively block the binding of Ubc9 to CRMP2, thereby inhibiting CRMP2 SUMOylation [35]. In neuronal cells, t-CSM significantly reduces the current density and membrane trafficking of Nav1.7; in neuropathic pain models, it can reverse mechanical and thermal hyperalgesia without motor dysfunction or sedation [35]. Notably, t-CSM has no effect on visceral pain, indicating its selective action [35].

### 6.3. Gene Silencing and Editing Technologies

Gene therapy offers a highly promising strategy for the long-term management of chronic pain. In contrast to conventional medications, its objective is to achieve sustained analgesia through a single intervention that induces lasting alterations in the expression or function of specific pain-related genes (such as CRMP2 and Nav1.7) at the source—for instance, in sensory neurons of the dorsal root ganglion. In recent years, significant progress has been made in technologies based on gene silencing and gene editing [49].

RNA Interference (RNAi): Small interfering RNA (siRNA) or short hairpin RNA (shRNA) targeting CRMP2 mRNA can induce its degradation. Studies have shown that intrathecal injection of CRMP2-specific siRNA can significantly reduce the CRMP2 protein level and effectively alleviate neuropathic pain [21].

Antisense Oligonucleotides (ASOs): Antisense oligonucleotides (ASOs) are another gene silencing tool that can bind to target mRNA and mediate its degradation through ribonuclease H. Due to their longer half-life and higher stability, ASOs have potential in pain treatment [49].

CRISPR/Cas9 Gene Editing: Theoretically, CRISPR/Cas9 technology can permanently knockout the CRMP2 gene or edit its functional domains in DRG sensory neurons [27]. However, due to safety issues caused by permanent manipulation and potential off-target effects, the clinical application of this technology in pain treatment is currently hindered, and it is mainly used as a basic research tool [27]. Upregulating the SENP1 through CRISPRa technology to enhance the deSUMOylation pathway can induce antinociception in the spinal nerve ligation model [25].

Packaging the CRS sequence into adeno-associated virus (AAV) for gene therapy delivery can significantly reduce Nav1.7 current in sensory neurons of rodents and non-human primates [33]. In models of nerve injury and chemotherapy-induced peripheral neuropathy, AAV-Nav1.7-CRS treatment can reverse and prevent mechanical allodynia and cold hyperalgesia without affecting motor function or physiological pain perception [33]. However, the long-term safety of this gene therapy still requires further evaluation [33].

### 6.4. Strategies Targeting Post-Translational Modifications of CRMP2

The function of CRMP2 is precisely regulated by a variety of post-translational modifications. Modulating these modifications provides an alternative approach for indirect intervention in CRMP2 function.

Phosphorylation Regulation: CRMP2 serves as a substrate for multiple kinases. GSK-3β can phosphorylate CRMP2 at Thr509/Thr514/Ser518, whereas ROCK can phosphorylate CRMP2 at Thr555, thereby reducing its tubulin-binding activity and affecting cytoskeletal regulation [50,51,52]. Although GSK-3β inhibitors such as TDZD-8 and ROCK inhibitors such as fasudil have demonstrated analgesic effects in preclinical pain models, these effects have mainly been linked to inflammatory, oxidative-stress, mitochondrial, or glial mechanisms rather than direct inhibition of CRMP2 phosphorylation [53,54,55,56]. In contrast, direct CRMP2 phosphorylation-targeted evidence in chronic pain is currently better supported by studies showing that Cdk5-mediated CRMP2 phosphorylation is necessary for neuropathic pain, phosphorylated CRMP2 regulates spinal nociceptive neurotransmission, (S)-lacosamide inhibits CRMP2 phosphorylation and reduces postoperative and neuropathic pain behaviors, and naringenin improves arthritic pain by inhibiting neuronal CRMP2 phosphorylation [21,24,26,40].

SUMOylation Regulation: Studies have shown that CRMP2 can be modified by the ubiquitin-like modifier SUMO (small ubiquitin-like modifier). This SUMOylation regulates CRMP2’s interaction with the voltage-gated sodium channel Nav1.7, thereby affecting neuronal excitability [7,34,35]. Therefore, targeting the SUMOylation/deSUMOylation cycle may become a new approach for the precise regulation of CRMP2 function, providing guidance for the development of small-molecule/peptide inhibitors (e.g., inhibitors targeting the SUMOylation pathway) [25,31,35].

### 6.5. Drug Repurposing: New Target Effects of Existing Drugs

By exploring the novel CRMP2-mediated mechanisms of approved drugs, drug repurposing offers significant potential for CRMP2-targeted pain treatment strategies and can accelerate clinical translation. Current research focuses primarily on the following drugs:

(S)-Lacosamide: Initially developed as an antiepileptic drug, studies have revealed that (S)-lacosamide inhibits CRMP2 phosphorylation and reduces pain behaviors; pharmacological inhibition of CRMP2 phosphorylation can also reduce presynaptic CaV2.2 and NaV1.7 localization in the spinal dorsal horn [40,41]. It has demonstrated favorable analgesic effects in models of neurofibromatosis type 1 (NF1)-associated pain, postoperative pain, and neuropathic pain, with its action dependent on specific sensory neuron subtypes [27,40].

Other Drug Screening Studies: High-throughput screening of existing drug libraries using known CRMP2-ion channel complex structures or cell-based phenotypic screening platforms may rapidly identify other “old drugs” with CRMP2 inhibitory activity. For example, the mechanism of action of certain diamide antiepileptic drugs may partially involve the CRMP2 pathway, although this requires further verification.

## 7. Concluding Remarks and Future Perspectives

Targeting CRMP2 shows great potential in the treatment of chronic pain. As a key molecular hub in pain signaling pathways, CRMP2 is involved in the initiation and maintenance of pain by regulating ion channel function, microtubule dynamics, and synaptic plasticity. This review systematically summarizes various CRMP2-targeted interventional strategies, including small-molecule inhibitors, peptide-based disruptors, gene technologies, and regulation of PTMs. These strategies have demonstrated significant analgesic efficacy and favorable safety profiles in preclinical models.

However, several conceptual and translational uncertainties remain. First, most current evidence is derived from rodent models, and it remains unclear whether the magnitude, timing, and cellular localization of CRMP2 dysregulation are conserved in human chronic pain syndromes. Second, although interventional studies targeting CRMP2 phosphorylation, SUMOylation, or CRMP2-ion channel interactions support a functional role for CRMP2 in pain regulation, it is still necessary to determine whether CRMP2 is a primary causal driver of chronic pain or, in some contexts, a downstream epiphenomenon of nerve injury, inflammation, or sustained neuronal activity. Third, CRMP2 PTMs may not be entirely pain-specific; they may also represent broader injury- or stress-related responses. Future studies should therefore include time-course analyses, cell-type-specific approaches, and rescue experiments to distinguish pain-driving CRMP2 changes from generic injury-associated alterations.

Despite persisting challenges in clinical translation, CRMP2 remains a promising target for next-generation non-opioid precision analgesics. Because CRMP2 is involved in neuronal polarity, axonal maintenance, cytoskeletal dynamics, and synaptic function, long-term CRMP2-targeted interventions should be carefully evaluated for potential effects on cognition, mood-related behaviors, psychiatric vulnerability, and axonal integrity. These considerations support the development of interaction-specific or pathway-selective CRMP2 modulators, such as agents targeting CRMP2–Ubc9, CRMP2–Nav1.7, or CRMP2–Cav2.2 interactions, rather than nonselective global CRMP2 inhibition.

Future research should focus on dissecting the CRMP2 interaction network, clarifying its causal contribution to chronic pain, optimizing clinical translation pathways, and actively exploring novel delivery systems, including nanoparticles and exosomes. Additional priorities include biomarker-based patient stratification, combination therapies with ion channel blockers or kinase inhibitors, and artificial intelligence (AI)-assisted drug discovery through virtual screening, drug repurposing, and multi-omics data integration. These efforts may ultimately support the development of safer and more effective CRMP2-based therapeutic strategies for patients with chronic pain.

## Figures and Tables

**Table 1:** Expression, Distribution, and Cellular Localization of CRMP2 Abnormal Regulation in Chronic Pain.

Pain Model	Time Point Examined	Region Examined	CRMP2 Alteration	Distribution and Cellular Localization	References
SNI-induced neuropathic pain (Sprague–Dawley rat)	Post-injury day 7	Lumbar spinal dorsal horn; DRG	CRMP2 SUMOylation ↑; total CRMP2 unchanged	Predominantly localized in dorsal horn; CRMP2 colocalized with NaV1.7 in DRG sensory neurons	[7]
SNI-induced neuropathic pain (Sprague–Dawley rat)	Post-injury day 12	Lumbar spinal dorsal horn; DRG	pCRMP2 (Ser522) ↑; total CRMP2 expression unchanged	Presynaptic sites of the spinal dorsal horn; DRG neurons	[21]
SNI-induced neuropathic pain (Sprague–Dawley rat)	Post-injury day 14	DRG	pCRMP2 (Ser522) ↑	pCRMP2 localized in NF200^+^, NaV1.8^+^/^−^ and IB4^+^/^−^ DRG sensory neurons	[22]
SNI-induced neuropathic pain (Sprague–Dawley rat)	Post-injury day 14	Contralateral insular cortex	CRMP2 ↑; pCRMP2 (Thr514) ↑; pCRMP2 (Ser522) unchanged	NA	[23]
SNI-induced neuropathic pain (Sprague–Dawley rat)	Post-injury day 10	Lumbar spinal dorsal horn	pCRMP2 (Ser522) ↑; total CRMP2 expression unchanged	Predominantly localized at presynaptic sites of the spinal dorsal horn; increased presynaptic pCRMP2 (Ser522) accompanied by enhanced presynaptic localization of CaV2.2 and NaV1.7	[24]
SNL-induced neuropathic pain (Sprague–Dawley rat)	Post-injury day 14	DRG neurons	CRMP2 SUMOylation reduced by SENP1 overexpression	NA	[25]
CIA-induced arthritic pain (Wistar rat)	PID21 and PID35 after final immunization	Lumbar spinal dorsal horn	pCRMP2 (Ser522) ↑; pCRMP2 (Tyr479) unchanged	CRMP2 colocalized with neurons	[26]
MIA-induced osteoarthritis pain (Sprague–Dawley rat)	2–3 weeks after MIA induction	Lumbar spinal dorsal horn	Total CRMP2 unchanged	No significant change in presynaptic CRMP2 localization	[8]
CRISPR/Cas9-mediated NF1-related pain (Sprague–Dawley rat)	7 days after final intrathecal Nf1 sgRNA injection	Lumbar spinal dorsal horn; DRG	pCRMP2 (Ser522) ↑	Spinal dorsal horn; DRG sensory neurons	[27]

↑: upregulation.

**Table 2:** Summary of the Therapeutic Potential of Targeting CRMP2 in Chronic Pain.

Target Category	Compound	Chemical Structure	Model	Treatment Strategy	Effect	Mechanism	References
SUMOylation Inhibitors	Compound 194	Small molecule	CCI Rats Osteoarthritic Rats Oxaliplatin-induced Mice	Intrathecal Injection, Oral	PWT ↑, PWL ↑ Prevents Pain Chronification	Inhibits CRMP2-Ubc9 interaction → Reduces Nav1.7 channel currents	[31,32,37,38]
	Intranasal 194	Same as above, intranasal formulation	Trigeminal Neuropathic Pain Model	Intranasal Administration	Pain Behaviors ↓	Modulates Nav1.7 in trigeminal pathways	[39]
Phosphorylation Inhibitors	(-)-Naringenin	Small molecule, Flavonoid (Natural Product)	Arthritic Pain Model	Intraperitoneal Injection	Arthritic Pain Behaviors ↓	Inhibits neuronal CRMP2 phosphorylation → Reverses spinal sensitization	[26]
	(S)-Lacosamide	Small molecule, Functionalized Amino Acid Derivative	Postoperative/Neuropathic Pain Models NF1-Related Pain Models	Systemic Administration	Pain Behaviors ↓	Inhibits Cdk5-mediated CRMP2 phosphorylation; reduces postoperative/neuropathic pain behaviors; reverses NF1-related pain-like behaviors	[27,40]
Protein Interaction Inhibitors (Cav2.2)	Myr-Tat-CBD3	Cell-penetrating peptide (N-myristoylated-TAT-CBD3)	Inflammatory/Postoperative pain models	Intrathecal Injection	Pain Behaviors ↓	Disrupts CRMP2–CaV2.2 interaction; reduces CaV2.2 trafficking and calcium-channel-dependent neurotransmitter release	[41]
Peptide Aptamers	TAT-CBD3-A6K	Cell-penetrating peptide aptamer (Modified CBD3 peptide)	Experimental Neuropathic Pain	Intrathecal Injection	Sustained relief of ongoing pain, Low abuse potential	Targets CRMP2, stabilizes inactive conformation, indirectly modulates ion channels	[42]
	Polyarginine-CRMP2 Peptide	Polyarginine-conjugated peptide	Peripheral Neuropathy Models	Intrathecal Injection	Pain-Related Behaviors ↓	Inhibits CRMP2 function, alleviates neuropathic pain	[43]
Gene Editing/Regulation	CRISPRa-SENP1	Gene therapy (CRISPR activation system)	SNL Model	Intrathecal Injection	Antinociception	Upregulates spinal SENP1 → Enhances deSUMOylation pathways	[25]
Other Interventions	Insular Cortex Stimulation	Physical Neuromodulation	Neuropathic Pain Model	Deep Brain Stimulation	Neuropathic Pain ↓	Alters CRMP2 expression (involved in synaptic plasticity)	[23]

↑: upregulation; ↓: alleviation.

## Data Availability

No new data were created or analyzed in this study.

## Abbreviations

CRMP: Collapsin Response Mediator Protein; CRMP2: Collapsin Response Mediator Protein 2; Nav1.7: voltage-gated sodium channel Nav1.7; Cav2.2: N-type voltage-gated calcium channel; PTMs: Post-Translational Modifications; CNS: Central Nervous System; CDK5: Cyclin-dependent kinase 5; GSK3β: Glycogen synthase kinase 3 beta; ROCK: Rho-associated protein kinase; SUMO: Small Ubiquitin-like Modifier; CCI: Chronic Constriction Injury; SNL: Spinal Nerve Ligation; SNI: Spared Nerve Injury; DRG: Dorsal Root Ganglion; VGCCs: Voltage-Gated Calcium Channels; VGSCs: Voltage-Gated Sodium Channels; NMDA: N-Methyl-D-Aspartate; NR2B: NMDA receptor subunit 2B; AMPA: α-amino-3-hydroxy-5-methyl-4-isoxazolepropionic acid; PPIs: Protein–Protein Interactions; h ERG: Human Ether-à-go-go-Related Gene; MCA: Mechanical Conflict Avoidance; TG: trigeminal ganglion; OA: Osteoarthritis; TAT-CBD3: TAT-fused CBD3 peptide; Myr-TAT-CBD3: N- myristoylatedTAT-CBD3 peptide; CBD3: CRMP2 Binding Domain 3; TAT-NF1: TAT-fused Neurofibromin-derived peptide; CSM: CRMP2 SUMOylation Motif; RNAi: RNA Interference; siRNA: small interfering RNA; ASOs: Antisense Oligonucleotides; SENP1: SUMO-specific protease 1; CRISPRa: CRISPR Activation; AAV: Adeno-Associated Virus; NF1: Neurofibromatosis type 1; AI: Artificial Intelligence.
